# P-1617. Clinical and microbiologic outcomes associated with implementation of MALDI-TOF on patients with anerobic, fungal, and non-lactose fermenting specimens

**DOI:** 10.1093/ofid/ofae631.1784

**Published:** 2025-01-29

**Authors:** Martina Boda, Nicholas J Mercuro, Kristina Rokas Connolly, Daniel Diekema, Christina Yen, Raymond Myatt

**Affiliations:** The Miriam Hospital, Coventry, Rhode Island; Maine Medical Center, Portland, ME; Maine Medical Center, Portland, ME; MaineHealth; Maine Medical Center, Portland, ME; NorDx, Scarborough, Maine

## Abstract

**Background:**

MALDI-TOF is a rapid-diagnostic technology that improves the speed and accuracy of organism identification. Using it to identify resistant pathogens and fastidious organisms is a high impact diagnostic stewardship opportunity. This study aims to evaluate its effect on the management of infections caused by non-lactose fermenting (NLF) Gram-negative rods (GNRs), anaerobes, and yeast from sterile sites.

**Figure 1.**
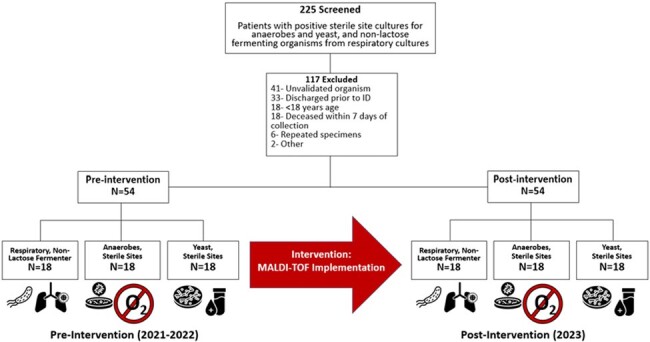
Screening Process

**Methods:**

This was a single center, quasi-experimental study of patients with NLF GNRs in respiratory specimens and anaerobes and/or yeast from sterile sites before and after the implementation of MALDI-TOF from 2021-2023. There were no changes in antimicrobial stewardship culture review or audit and feedback processes during the study period. The primary outcome was time to organism identification. Secondary endpoints were time to antimicrobial adjustment, inpatient antibiotic duration, and in-hospital mortality. Categorical descriptive data was expressed as percentages and as median/interquartile range (IQR) if continuous. Chi-squared/Fisher’s Exact test and Mann-Whitney-U were used as appropriate to compare endpoints between groups.

**Table 1.**
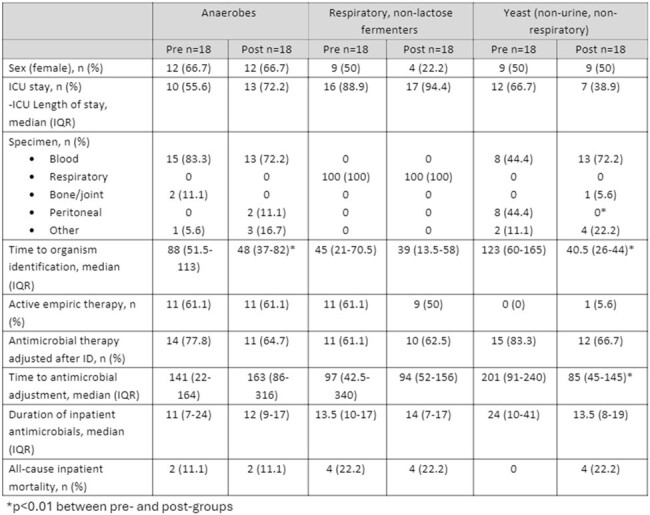
Patient characteristics and Secondary Endpoints by MALDI-TOF Identified Organism

**Results:**

Of 225 patients screened, 108 were included (Figure 1). The median Charlson score was 3 (1-5). The overall length of stay was 30 (13-54) days; 75 (70%) patients had an ICU stay with a median ICU length of stay of 17 (4-31) days. The most common organisms identified were Pseudomonas aeruginosa, Bacteroides spp, Candida albicans, and Nakaseomyces glabratus (formerly C. glabrata) (Figure 2). Time from Gram-stain to organism identification was reduced from 77 (41-119) hours to 41 (26-56) hours (p< 0.001) (Table 1). Time to antimicrobial adjustment and inpatient duration of antibiotics were not different between pre- and post- implementation of MALDI-TOF for infections caused by anaerobic and NLF GNRs but was reduced in patients with yeast in sterile sites. There was no difference in in-hospital mortality.

**Figure 2.**
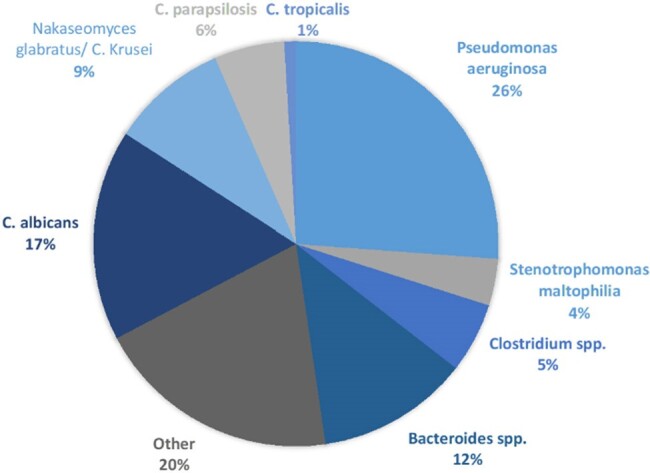
Percentages of Organisms Identified by MALDI-TOF

**Conclusion:**

Implementation of MALDI-TOF for identification of these organisms improved culture processing turn-around time, antimicrobial optimization, particularly for yeast, and stewardship efforts at our institution.

**Disclosures:**

**Daniel Diekema, MD, MS, D(ABMM)**, Affinity Biosensors: Grant/Research Support|bioMerieux, Inc: Grant/Research Support

